# Structural Features and Toxicity of α-Synuclein Oligomers Grown in the Presence of DOPAC

**DOI:** 10.3390/ijms22116008

**Published:** 2021-06-02

**Authors:** Luana Palazzi, Benedetta Fongaro, Manuela Leri, Laura Acquasaliente, Massimo Stefani, Monica Bucciantini, Patrizia Polverino de Laureto

**Affiliations:** 1 Department of Pharmaceutical and Pharmacological Sciences, University of Padova, 35131 Padova, Italy; luana.palazzi@unipd.it (L.P.); benedetta.fongaro@unipd.it (B.F.); laura.acquasaliente@unipd.it (L.A.); 2 Department of Biomedical, Experimental and Clinical Sciences, University of Firenze, 50134 Firenze, Italy; manuela.leri@unifi.it (M.L.); massimo.stefani@unifi.it (M.S.); monica.bucciantini@unifi.it (M.B.)

**Keywords:** α-synuclein aggregation inhibition, fibril inhibition, DOPAC, Parkinson’s disease, protein oligomerization, oligomer toxicity, autophagy

## Abstract

The interplay between α-synuclein and dopamine derivatives is associated with oxidative stress-dependent neurodegeneration in Parkinson’s disease (PD). The formation in the dopaminergic neurons of intraneuronal inclusions containing aggregates of α-synuclein is a typical hallmark of PD. Even though the biochemical events underlying the aberrant aggregation of α-synuclein are not completely understood, strong evidence correlates this process with the levels of dopamine metabolites. In vitro, 3,4-dihydroxyphenylacetaldehyde (DOPAL) and the other two metabolites, 3,4-dihydroxyphenylacetic acid (DOPAC) and 3,4-dihydroxyphenylethanol (DOPET), share the property to inhibit the growth of mature amyloid fibrils of α-synuclein. Although this effect occurs with the formation of differently toxic products, the molecular basis of this inhibition is still unclear. Here, we provide information on the effect of DOPAC on the aggregation properties of α-synuclein and its ability to interact with membranes. DOPAC inhibits α-synuclein aggregation, stabilizing monomer and inducing the formation of dimers and trimers. DOPAC-induced oligomers did not undergo conformational transition in the presence of membranes, and penetrated the cell, where they triggered autophagic processes. Cellular assays showed that DOPAC reduced cytotoxicity and ROS production induced by α-synuclein aggregates. Our findings show that the early radicals resulting from DOPAC autoxidation produced covalent modifications of the protein, which were not by themselves a primary cause of either fibrillation or membrane binding inhibition. These findings are discussed in the light of the potential mechanism of DOPAC protection against the toxicity of α-synuclein aggregates to better understand protein and catecholamine biology and to eventually suggest a scaffold that can help in the design of candidate molecules able to interfere in α-synuclein aggregation.

## 1. Introduction

**Figure 1:**
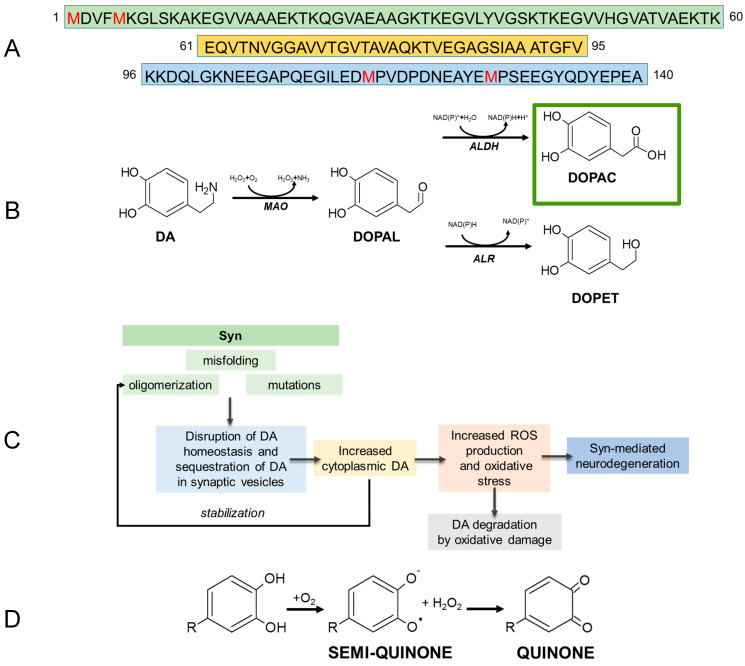
Amino acid sequence of Syn. The three structural domains (1–60, green; 65–95, yellow; 96–140, light blue) are shown. Methionine residues are colored in red (**A**). Main DOPAL metabolites formed in vivo through enzymatic reaction. The DOPAC structure is highlighted in green. MAO, ALDH, ALR are the enzymes involved in the reactions, monoamine oxidase, aldehyde dehydrogenases, aldose reductase (**B**). Simplified scheme of a possible correlation between catechols and neurodegeneration (**C**). Autoxidation mechanism of catechols with formation of semi-quinone, quinone and H_2_O_2_ in aerobic environment (**D**).

Parkinson’s disease (PD) is a multifactorial age-related disorder, whose typical hallmark is the presence of cytoplasmic inclusions known as Lewy bodies (LB) in the dopaminergic neurons of the substantia nigra pars compacta [1,2]. It is considered the second most common aging-associated neurodegenerative disease. The material accounting for most of the LB content is a mesh of amyloid β-sheet-rich fibrils arising from the polymerization of α-synuclein (Syn), a relatively small protein (14.5-kDa), mainly located at the neuron presynaptic terminal [3]. Intermediate transient oligomeric species are generated early in the pathway of Syn fibrillar aggregation, and these species, rather than fibrils, are supposed to be the toxic entities responsible for neuron sufferance and death [4,5,6]. Syn is classified as natively unfolded, but it acquires α-helical secondary structure in the presence of anionic membranes and lipids following a conformational change mediated by the first 100 *N*-terminal residues [7,8,9]. The sequence of human Syn, shown in Figure 1A, is conventionally divided into three domains. The *N*-terminal region (residues 1–60) contains imperfect 11-amino acid sequence repeats with the highly conserved KTKEGV motif. The 61–95 residues are hydrophobic and form the amyloidogenic NAC (non-amyloid-β component) domain, responsible for protein aggregation [10]. The C-terminal region (residues 96–140) is both hydrophilic, because of the presence of 14 acidic residues, and disordered, due to the content of several proline residues; this region binds metals [11] and could modulate Syn amyloidogenicity [12,13]. The biochemical events that determine the formation of Syn fibrils in vivo are not completely understood, though compelling evidence suggests a significant link between Syn aggregation and intracellular levels of neuronal catechols, such as dopamine (DA) and its metabolites, particularly 3,4-dihydroxyphenylacetaldehyde (DOPAL) (Figure 1B,C). DA and its metabolites are involved in the condition of oxidative stress [14] and mitochondrial dysfunction [15] associated with PD, and can modulate Syn aggregation both in vitro and in vivo [2,16,17].

Previous research has suggested that DA and derivatives can be considered as two-faced signal molecules: both essential elements for normal neural function, but also key factors in oxidative stress-driven neurodegeneration. In particular, the strong correlation between long-term DOPAL build-up in neurons and the growth of Syn aggregates has contributed to the “catecholaldehyde hypothesis”, to explain the selective loss of nigrostriatal dopaminergic neurons in PD [18,19]. In animal models and human cultured cells, an increase of cytosolic DA and DOPAL results in the appearance of Syn oligomers [20,21,22] that cannot be converted to amyloid fibrils [23,24]. These oligomers are cytotoxic at the concentrations found post-mortem in PD patients [25] possibly by affecting membrane functionality [26]. It has been proposed that DA is a major source of the oxidative stress associated with early Syn-mediated neurodegeneration, and its cytosolic increase is responsible for the selective loss of dopaminergic neurons. Experimental evidence points out how A30P Syn mutant can increase cytoplasmic DA by modifying the activities of molecular components responsible for its synthesis, storage or uptake, thus being responsible for the perturbation of cytosolic catecholamine homeostasis [27]. Very reduced information is available about the two DA metabolites, 3,4-dihydroxyphenylacetic acid (DOPAC) and 3,4-dihydroxyphenylathanol (DOPET) (Figure 1B). In vivo, DOPAC arises from the enzymatic oxidation of DOPAL and then is rapidly eliminated from the neurons. It is much less reactive for the presence of a carboxyl moiety, but as its precursor, in the presence of O_2_, it auto-oxidizes producing H_2_O_2_ [28], in turn, an oxidizing agent and ROS generator (Figure 1D). DOPAL interacts with Syn forming covalent adducts with Lys residues that hinder protein interaction with cell membranes and leads to the formation of small oligomers that potently inhibit the growth of mature amyloid fibrils [29]. In vitro, at low concentrations, DOPAC interacts non-covalently with monomeric Syn. The oxidized form (the quinone) of DOPAC is responsible for the oxidation of the methionine residues of Syn, probably following H_2_O_2_ production; at higher concentrations, DOPAC can form covalent adducts with the protein in analogy to DOPAL [30]. 

In a previous study, we investigated in vitro the activity of DOPET, another product of DOPAL metabolism, on Syn fibrillation [31]. DOPET proved to be an efficient inhibitor of the formation of cross β-sheet structures of Syn, inducing the formation of stable off-pathway oligomers. Furthermore, when added to the aggregation mixture, it likely formed covalent bonds with Syn, through Michael addition reactions. The cytotoxicity of DOPET-induced oligomers was tested on cultured neuroblastoma cells; these oligomers were non-toxic, differently to Syn on-pathway oligomers and fibrils, which caused ROS formation and decreased cell viability [31]. The molecular mechanism underlying inhibition of Syn fibrillation by catechols is a question still debated. Apparently, all catechols inhibit the conversion to fibrils of monomeric Syn while inducing oligomer formation. A common paradigm is provided by the catechols-induced covalent modifications of Syn, ranging from the oxidation of Met residues [32] to the formation of covalent adducts on lysine residues [26,29]. DOPAL could also interact with Syn in a non-covalent manner, by binding to the 125–129 sequence (YEMPS) in the C-terminal region [33]. So far, the role of protein oxidation in Syn aggregation and aggregate toxicity is not completely understood, although morphological and biological differences in the type of produced oligomers have been described [22]. Better knowledge of the molecular mechanism underlying the inhibition of Syn fibrillation by catechols is needed for several reasons: (i.) the characterization of the resulting oligomers and their intrinsic activity is still incomplete; (ii.) the reason why these aggregates accumulate in vivo is not known. Hampering Syn aggregation represents an important field of investigation in pharmaceutical and pharmacological ambits. To date, all drugs used for PD treatment are palliative: they do not prevent disease progression, but only relieve symptoms to improve the life quality of patients. Consequently, finding novel, druggable targets for therapeutic intervention remains a top priority to prevent or delay disease progression. A better knowledge of the molecular basis of the interaction of Syn with compounds able to inhibit its fibrillation might help to design new molecules for targeted therapy.

In the present work, we show that similarly to DOPAL and DOPET, DOPAC inhibits Syn aggregation by stabilizing the monomeric form of the protein and, at a lesser extent, inducing the growth of oligomeric species, particularly dimers and trimers, that do not proceed further to fibrils. The early radical products of DOPAC autoxidation react with Syn, producing covalent modifications on the protein, including Met oxidation and Michael addition reactions involving Lys residues. This chemical modification of Lys occurs to a very limited extent, while Met oxidation involves all the four Met residues present in Syn sequence. However, our findings show that Met oxidation does not inhibit, by itself, fibril formation and is not the only cause of oligomerization. In fact, a population of Syn off-pathway oligomers was detectable when Syn oxidation was hampered by using catalase that scavenges the H_2_O_2_ generated by DOPAC autoxidation. Our data evidence that the inherent features of Syn oligomers generated by DOPAC were responsible for the overall observed biological and biophysical effects, whereas the chemical modifications were important but not essential. Moreover, these Syn aggregates manifested a low propensity to undergo conformational transition in the presence of cellular membranes. Finally, cellular assays showed that Syn aggregates grown in the presence of DOPAC exhibited reduced cytotoxicity and ROS production. Intriguingly, DOPAC/Syn oligomers activated lysosomal activity favoring the clearance of Syn aggregates and attenuating Syn build-up within Syn-exposed cells. Overall, our results contribute to providing a unifying view of Syn species induced by DOPAC and useful information for the future design of PD-modifying therapies.

## 2. Results

### 2.1. DOPAC Affects Amyloid Aggregation of α-Synuclein

**Figure 2:**
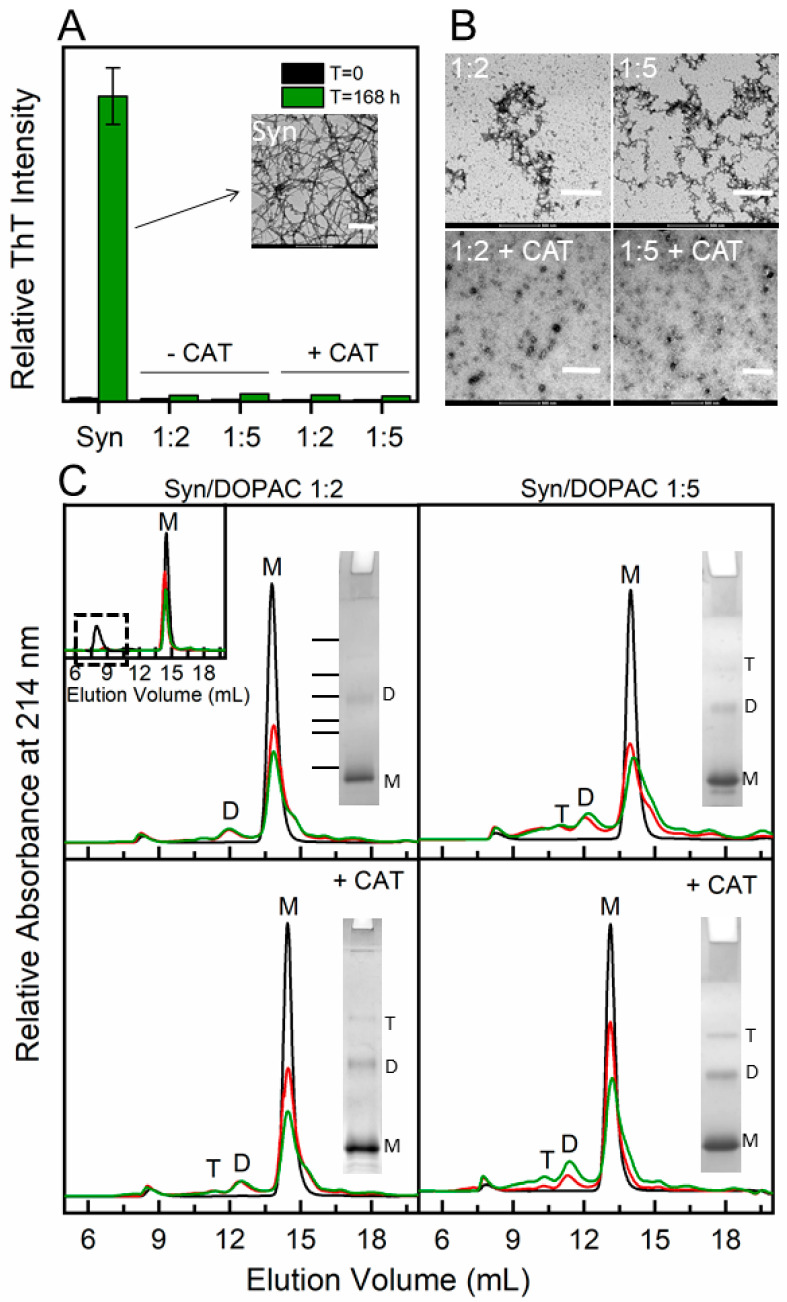
Aggregation process of Syn in the presence of DOPAC monitored by ThT assay (**A**), TEM (**B**) and SEC (**C**). The intensity of ThT fluorescence at 485 nm, after excitation at 440 nm for Syn/DOPAC (1:2 and 1:5) in the absence (−) and in the presence (+) of catalase (CAT) was reported in comparison to Syn alone after 0 and 168 h of incubation. Inset: Syn fibrils by TEM after 168 h of incubation (**A**). TEM pictures of Syn/DOPAC 1:2 and 1:5 samples after 168 h of incubation in the absence and in the presence (+) of CAT (B) Scale bar: 500–200 nm. SEC profiles of Syn/DOPAC (1:2 and 1:5) samples after 0 (black), 72 (red) and 168 (green) h of incubation in the absence (up) and in the presence (down) of CAT. Inset up: SEC profiles of Syn alone. Zooming (×100) shows the peak at 8 mL elution volume. SDS-PAGE lines of the mixture of Syn/DOPAC relative to 168 h were also shown (**C**). The lines close to the gel bands indicate the position of proteins used as marker of molecular weight (66, 45, 36, 29, 24 and 20 kDa).

DOPAC shares with other catechols the capability to alter the amyloidogenic process that leads to the growth of Syn fibrils [30]. Syn aggregation was studied by evaluating how different molar ratios of DOPAC affect the fibrillation process. A 70 μM Syn solution was mixed with 140 μM or 350 μM DOPAC (1:2 and 1:5, respectively). In a previous study, a 1:1.2 Syn:DOPAC molecular ratio completely inhibited fibril formation [30]. Higher ratios were used to find a correlation between DOPAC dose and its effect to induce oligomerization. These mixtures were incubated at 37 °C under shaking to promote Syn fibrillation, assessed by Thioflavin T (ThT) assay, TEM imaging, size exclusion chromatography (SEC) and SDS-PAGE (Figure 2). The intensity of ThT fluorescence emission at 485 nm of Syn and 1:2 and 1:5 Syn/DOPAC samples (Figure 2A) collected at different aggregation times was recorded. Only Syn alone displayed a significant increase of fluorescence that indicated the presence of amyloid aggregates (Figure 2A, inset), confirmed by the TEM images, showing fibrillar components typical of PD [34]. However, DOPAC-containing samples did not display any significant emission at 485 nm (Figure 2A), indicating the absence of fibrils, as confirmed by TEM images, where only amorphous aggregates, quite different from those formed by Syn alone, were present (Figure 2B). 

Then, the aggregation mixtures were analyzed by SEC to assess the aggregation state of Syn in the presence of DOPAC (Figure 2C). Samples were collected from the aggregation mixture at steps where mainly transient oligomers (48–72 h) and fibrils (168–192 h) were populated (Figure S1). The samples were ultra-centrifuged before loading onto the column, to remove insoluble particles from the aggregation mixture. Under these conditions, after the ultracentrifugation step, a pellet was recovered in Syn sample incubated in the absence of DOPAC (Figure 2C, inset) only after prolonged incubation (168 h), whereas no pellet was found in Syn/DOPAC samples, suggesting the formation of soluble species during incubation. The SEC analysis of Syn sample before incubation showed a main peak at 14 mL elution volume corresponding to a value of 56 kDa in the calibration curve and hence to monomeric Syn (M), (Figure S2). Upon incubation under aggregation conditions, the intensity of the peak corresponding to monomeric Syn was greatly reduced due to fibril growth. When Syn/DOPAC samples were investigated (Figure 2C), a first peak eluting at 8.0 mL was present at all DOPAC concentrations used. This peak corresponded to Syn amorphous aggregates arising during protein handling, as in the control (Figure 2C, inset) and its intensity increased over time, suggesting that high-molecular-weight species are stabilized by the incubation with DOPAC, while this peak disappeared in Syn samples incubated alone for 168 h. Furthermore, between the first peak and the monomeric Syn peak (M, 14 mL), peaks identifiable as Syn trimer (T, 11 mL) and Syn dimer (D, 12 mL) were seen. In addition, in this range of elution volumes, the baseline increasingly shifted upwards proportionally to the molar concentration of DOPAC. After monomeric Syn, different species, probably degraded forms, were eluted. These findings indicate that, in the presence of DOPAC, Syn produces several aggregated species that are not present when Syn is incubated alone; these species elute before monomeric Syn during the isocratic run, indicating a greater hydrodynamic volume. The drop-in intensity of the monomeric Syn peak in all samples aggregated 168 h is due to fibrils formation in the case of Syn alone, and to the formation of other aggregates in the presence of DOPAC.

DOPAC, as a catechol, undergoes auto-oxidation (Figure 1D), leading to the formation of o-quinone derivatives and H_2_O_2_, with the ensuing oxidation of the four Met residues in Syn, as confirmed by MS analysis (Table 1). Oxidation of Met residues inhibits remarkably Syn fibrillation in vitro and favors the formation of relatively stable oligomers [32]. For this reason, to prevent Met oxidation and to remove its contribution in the protein oligomerization, a set of experiments was performed to scavenge the formation of H_2_O_2_ by adding the enzyme catalase (CAT) to the aggregation mixture. In a previous study, we showed that CAT did not affect either the kinetics of Syn fibril formation or the properties of catechol-containing molecules [31]. Accordingly, we established by ThT assay and TEM imaging the capability of DOPAC to affect Syn fibrillation when CAT was present in the aggregation mixture (Figure 2A,B). In agreement with the results obtained in the absence of CAT, we found that, under these conditions, the fibrils were completely absent in the samples and the fluorescence did not increase. The collected samples were analyzed by SEC (Figure 2C) and the chromatograms were comparable to those obtained in the absence of CAT, suggesting similar hydrodynamic volumes for the aggregated species grown in the presence of DOPAC in the two sets of experiments. The drop in the intensity of the peak corresponding to monomeric Syn in all samples after incubation is related to fibrils growth in the case of Syn alone, and to the formation of other aggregates, mostly dimer and trimer, in the presence of DOPAC. In the presence of DOPAC + CAT, more dimeric and trimeric species were produced with respect to those arising in the presence of DOPAC, and the morphology of the forms produced under the two conditions was quite different. In fact, the aggregates grown in the absence of CAT appeared amorphous, while TEM analysis showed that in the presence CAT, Syn assembled into ring-like oligomers (Figure 2B). The dimensions of these spherical oligomers were not homogenous and the outer diameters ranged between 50–70 nm with an average size of ~61 nm. The TEM measurements were conducted on the Syn/DOPAC mixture, and the morphology was similar to previously described [35]. The products arising in Syn/DOPAC mixtures in the presence or in the absence of CAT incubated 168 h were further characterized by SDS-PAGE showing dimeric and trimeric species (Figure 2C, inset). The band of monomeric Syn was slightly less intense in Syn/DOPAC 1:5 than in Syn/DOPAC 1:2 samples, while the oligomeric forms of Syn were prevalent. 

### 2.2. α-Synuclein Undergoes Chemical Modifications and Conformational Transition to Oligomeric Forms in the Presence of DOPAC

**Figure 3:**
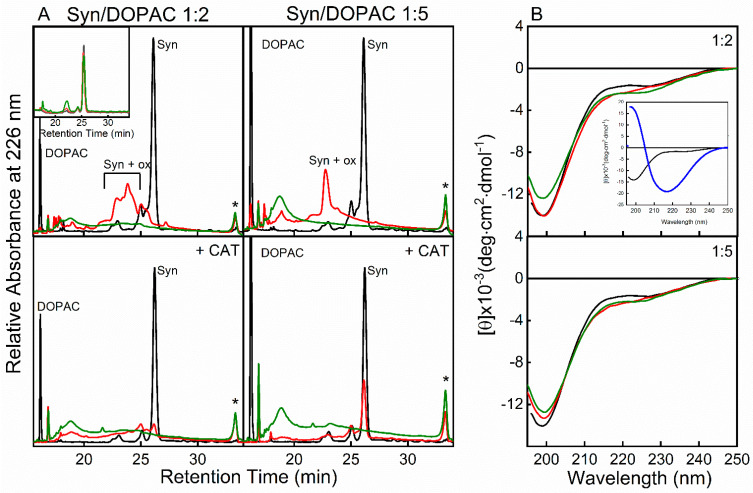
RP-HPLC analysis. The chromatograms of Syn/DOPAC (1:2 and 1:5) samples after 0 (black), 48 (red) and 168 (green) h of incubation in the absence (up) and in the presence (down) of CAT. Inset: RP-HPLC profiles of Syn samples taken after 0 (black), 48 (red), 168 (green) h of incubation. * indicates the oligomeric species (**A**). Far-UV CD spectra of Syn (black line) and Syn/DOPAC 1:2 (up) and 1:5 (down) samples, after 0 (black), 168 h (red) and 168 h in the presence of CAT (green) h of incubation. Inset: Far-UV CD spectra of Syn (black) and Syn after 168 h of incubation after shaking (blue) (**B**).

Figure 3 shows the RP-HPLC analysis of Syn samples collected from the aggregation mixture in the presence of DOPAC. It appears that Syn chromatographic profile was not affected by DOPAC before sample incubation, and the RP-HPLC chromatogram displayed a peak at ∼25 min identical to that recorded in the control (Figure 3A, inset); the intensity of the peak relative to DOPAC at ~16 min varied based on its concentration in the mixture. However, in the presence of DOPAC, Syn peak area decreased and new peaks were detected in the chromatograms. ESI-Q-TOF-MS analysis of the RP-HPLC fractions eluting before Syn (Table 1) shows that Met residues had been oxidized. In the presence of higher concentration of DOPAC (350 μM), all Met residues were in the oxidized form after 48 h of incubation (Figure 3A, red lines). The decrease of Syn peak intensity and the lack of accumulation of oxidized Syn in the aggregation mixture were balanced by the appearance of a new species eluting at 33.2 min in the chromatograms. The intensity of this species matched DOPAC concentration and incubation time (Figure 3A, *). This species was characterized by SDS-PAGE (not shown) and MS analysis (Table 1). In agreement with previous results [31,37], different species populated this fraction, comprising aggregated and chemically modified Syn. For confirmation, the SEC fractions corresponding to dimeric and trimeric (D + T) species were reloaded in RP-HPLC and eluted at 33.2 min retention time (RT) (Figure S3). Moreover, in RP-HPLC the SEC fraction corresponding to the monomer (M) was resolved into a peak at 25.1 min RT, where also native Syn elutes, and to another peak at 33.2 min RT (Figure S3). The mass spectrum of the peak at 33.2 min RT was not well resolved and indicated the presence of oxidations (+16 Da) and another chemical modification resulting in a mass increase of 121 Da (Table 1). The latter mass increase corresponds to the formation of a covalent adduct, resulting from a Michael addition reaction, involving likely the ε-amino group of Lys residues and the aromatic ring of DOPAC [29], whose proposed structure is shown in Table 1. When we analyzed by MS Syn/DOPAC samples incubated 48 h in the presence of CAT, we found that the protein was not oxidized (Table 1), confirming that the presence of CAT in the aggregation mixture avoids Syn oxidation by H_2_O_2_. However, the 33.2 min species was still present, and the intensity of this fraction raised as far as the incubation was prolonged and the amount of DOPAC was increased. The mass spectrum of this fraction contained several poorly resolved signals, indicative of aggregated species. It also showed the presence of chemical modifications in Syn sequence due to covalent adduct with an increase of the mass of 121 Da. Samples corresponding to SEC fractions M and D + T were also analyzed by native MS, which enables the study of non-covalent protein–protein and protein–ligand complexes under native conditions. This analysis showed the presence of polymeric DOPAC species in both samples and roughly indicated the entity of the covalent modification (~6%), as calculated based on the area beneath the peaks of Syn and Syn + 121 (Figure S4). 

The interaction between Syn and DOPAC was also investigated by far-UV circular dichroism (CD) (Figure 3B). DOPAC did not induce conformational changes in Syn in any of the used experimental conditions, and Syn spectrum was typical of a completely disordered structure (black line). The CD spectra revealed the presence of a random conformation even upon prolonged incubation times (red line). The presence of CAT (green line) did not apparently affect the structural properties of Syn/DOPAC samples, resulting only in a very slight change in the shape of Syn spectra. In the inset, as a control, the far-UV CD of Syn at T = 0 (black line) and of Syn incubated 168 h at 37 °C under mixing (blue line) are reported. The last spectrum, containing a minimum at ~218 nm, showed the presence of β-sheet structure in Syn, indicative of fibril formation.

### 2.3. Monomeric, Dimeric, and Trimeric Syn Exhibit Different Susceptibility to Proteolysis

**Figure 4:**
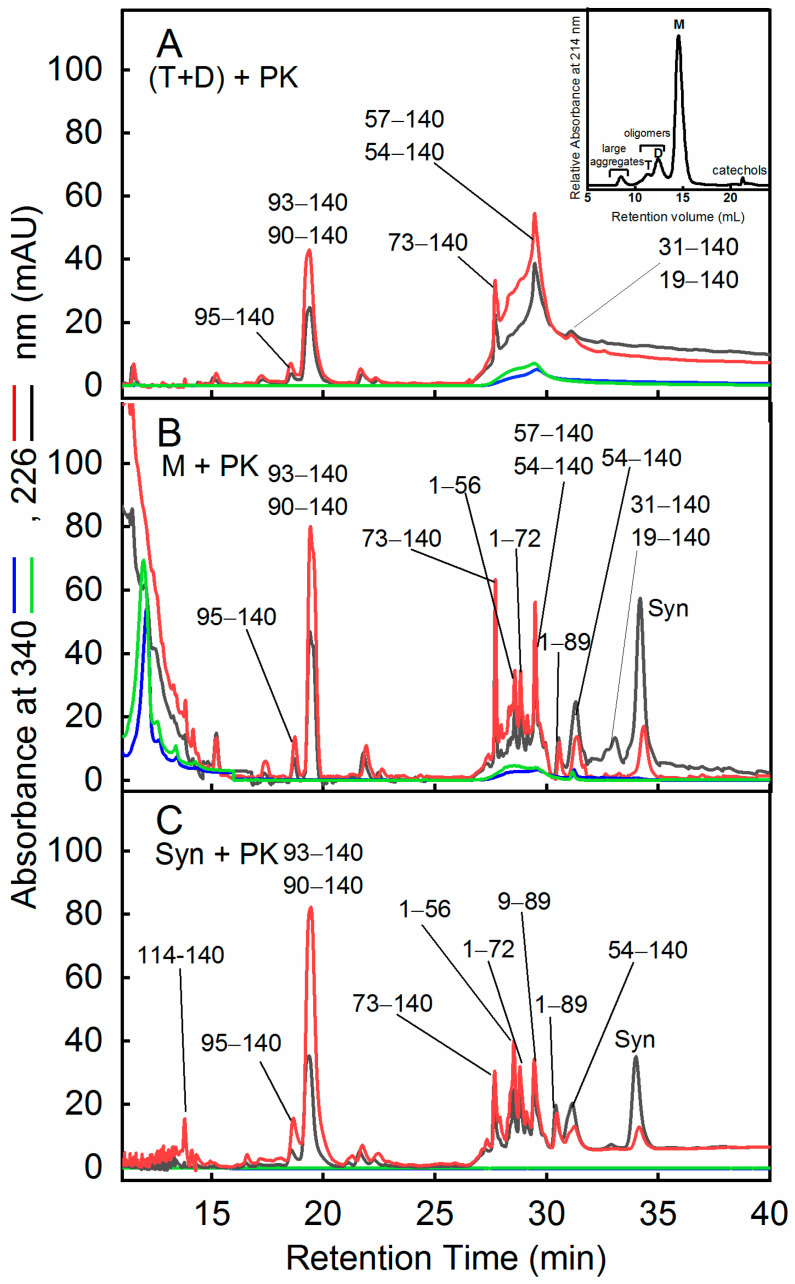
Proteolytic mapping of oligomeric species of Syn by proteinase K (PK). RP-HPLC chromatograms of the proteolytic mixtures of the SEC (inset) fractions corresponding to dimer, D and trimer, T (**A**) and monomer, M (**B**). Aliquots were collected from the reaction mixtures after 5 (black or blue lines) and 10 (red or green lines) minutes of incubation with the protease and analyzed monitoring the signals at 226 (black and red lines) and 340 (blue and green lines) nm. As a control, the proteolytic mixture of Syn with PK, obtained under the same conditions, was shown (**C**). Proteolysis experiments were conducted by using an enzyme to protein ratio of 1:1000. The RP-HPLC conditions were reported in the experimental section. The identity of the species was assessed by mass spectrometry (Table 2). Inset: SEC profile referring to the analysis of Syn/DOPAC (1:5) samples after 48 h of incubation in the presence of CAT.

Next, for a better characterization of Syn monomeric, dimeric, and trimeric species and to eventually identify regions of the protein interacting with DOPAC molecules, the stability to protease treatment was evaluated. We expected that protein regions engaged in an interaction with DOPAC would display a reduced tendency to be cleaved by proteases. To do this, the SEC fractions corresponding to the oligomers (D + T) and to monomer (M) (Figure 4A, inset) were treated with proteinase K (PK), an enzyme with wide specificity [38], at a 1:1000 enzyme to protein ratio (Figure 4). It is expected that the cleavage sites are not determined by the amino acid sequence; rather, they are dictated by the conformational features and flexibility of the polypeptide chain [39,40]. To simplify the interpretation by RP-HPLC and MS of the reaction products, we analyzed the aggregates grown in the presence of DOPAC and CAT. Syn/PK proteolytic mixtures incubated under the same conditions were reported as a control. The results relative to the proteolytic mixtures obtained by incubation with PK for 5 min and 10 min are reported in Figure 4 and Table 2. The chromatographic profiles obtained at 340 nm refer to DOPAC-bound species or to DOPAC derivatives (Figure S5). 

PK cleaves Syn and the species obtained in the presence of DOPAC, producing similar fragments at different extents. Syn, as an unfolded protein, undergoes very fast enzymatic fragmentation and, as previously observed [31,37], the peak relative to the intact protein decreases in intensity in the proteolytic mixture within few minutes of incubation with the protease. In the case of the reaction involving monomeric Syn form SEC (M), proteolysis appears slightly slower than that of control Syn and larger fragments (31–140; 19–140) were found in the mixture. These fragments were detected also in the proteolysis mixture of the oligomers (D + T) (Figure 4A and Figure S6). The presence of these large fragments reveals a protease-resistant core in the oligomers formed with DOPAC. Indeed, such peptides were previously found in the case of proteolysis of Syn fibrils [31]. Of note, in the proteolysis mixture of M, the 54–140 and 57–140 fragments eluted in two different chromatographic fractions, at 29.5 min and 31.1 min (Table 2). This suggests that M resulted from two populations coexisting at equilibrium. RP-HPLC analysis of M (Figure S3) shows two main fractions at two RTs. The fraction eluting earlier displayed the same retention time of Syn, whereas the other eluted later as a very hydrophobic and large species, appeared more compact or at least more protected from proteolysis. The peptides derived from the proteolysis of dimer and trimers (D + T), eluting with an RT of 27–35 min (Figure 4A; Table 2), appeared as not resolved indicating that they are strongly aggregated or at least covalently linked. Differently from the peptides obtained from proteolysis of control Syn, a group of peptides was absorbed at 340 nm, indicating the presence of the chemical modification induced by DOPAC or a strong interaction with the latter. No fragment starting from residue 73 or 90 absorbed at 340 nm, indicating the absence of chemical modifications in this region, whereas the species 1–56, 1–72, and 54,57–140 absorbed at this wavelength; the absorption intensity was similar in M and D + T samples and seemed to involve a relatively small fraction of Syn molecules. Another difference between the two chromatographic profiles of the proteolytic mixture of M was the presence of DOPAC signals at elution times <15 min. This finding suggests that M species, differently from D + T, released DOPAC upon fragmentation by PK. In conclusion, the species generated in the presence of DOPAC appear globally more compact with a slight, yet significantly, reduced susceptibility to proteolysis. The increased resistance to proteolysis was evaluated considering the signals relative to intact Syn not cleaved by PK. Indeed, by integrating the area beneath the peaks of intact Syn (RT 34.5 min) in the chromatograms reported in Figure 4B,C, after 5-min reaction with PK, a 2.3-fold increased resistance to proteolysis in the presence of DOPAC was estimated.

### 2.4. DOPAC Decreases the Interaction of α-Synuclein with Synthetic Membranes

**Figure 5:**
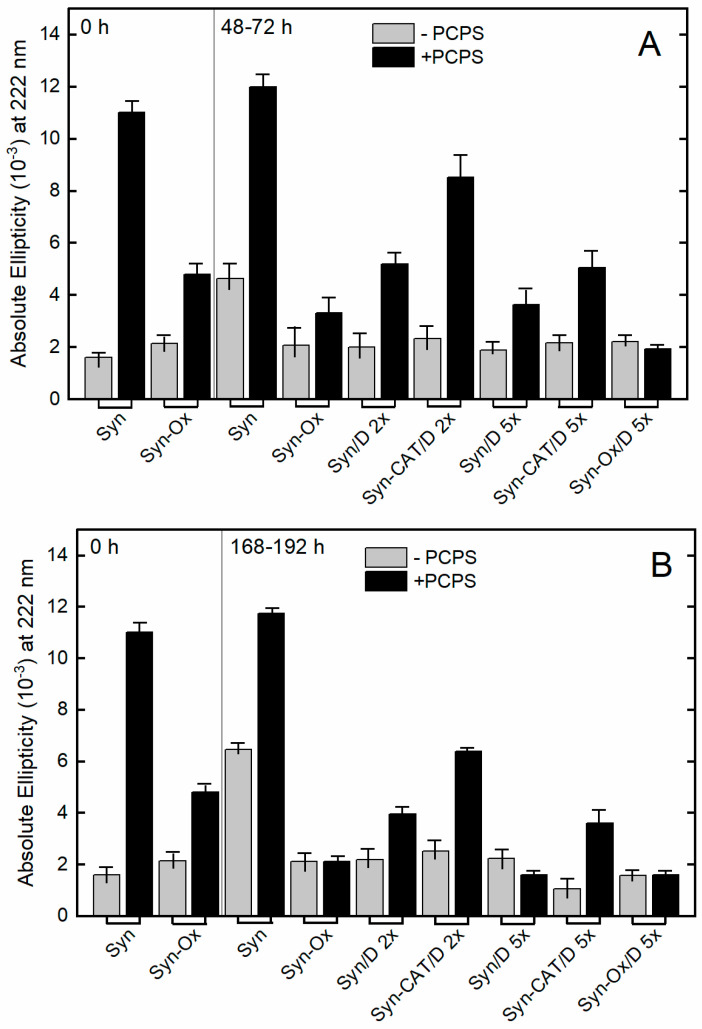
Syn membrane interaction probed by far-UV CD. Ellipticity at 222 nm of Syn samples recorded under upon incubation at 0, 48–72 (**A**), 168–192 h (**B**) in the presence (black columns) and in the absence (grey columns) of PC:PS (1:1) liposomes, in the presence and in absence of DOPAC and the CAT. In the labels, D indicates DOPAC and 2x–5x indicates the catechol/protein ratio. The ellipticity of Syn, Syn in the presence of CAT (Syn-CAT) and oxidized Syn (Syn-Ox) were reported in the same conditions as a control. Error bars indicate the error derived from two sets of experiments conducted at different times of incubation.

Syn interacts with lipid vesicles that contain negatively charged polar head groups, such as PC:PS liposomes, undergoing α-helix transition [7,8,9]. We investigated the effect of DOPAC on the appearance of α-helix in Syn exposed to negative membrane by evaluating the protein conformational transition in the presence of PC:PS liposomes by monitoring the signal in the far-UV CD at 222 nm, under different experimental conditions (Figure 5). The measurements involved Syn samples collected during incubation in the presence or in the absence of DOPAC (1:2 and 1:5 Syn/DOPAC) and of CAT at the growth (48–72 h) and plateau (168–192 h) phases. As a control, the conformational modifications of Syn and oxidized Syn (Syn-Ox) were monitored under the same conditions. As reported in Figure 3B, DOPAC did not affect the random structure of Syn that remained unstructured at the end of the incubation. After 48–72 h of incubation in the presence of PC:PS, the ellipticity at 222 nm of Syn/DOPAC 1:2 sample (Syn/D 2X) was roughly half that of Syn sample without DOPAC. Such a reduction was more intense for the samples containing a 1:5 Syn/DOPAC ratio (Figure 5A). When the time of incubation in the presence of DOPAC was prolonged (168–192 h), the CD signals at 222 nm further decreased in the samples containing both Syn/DOPAC ratios (Figure 5B). These findings indicate that DOPAC clearly reduced the interaction with PC:PS liposomes of Syn or of Syn aggregated species generated in its presence, in a concentration-dependent and time-dependent manner. This behavior was opposed to that recorded in the presence of Syn alone. As a control, the ellipticity of Syn samples incubated with PC:PS liposomes was reported. It maintained the same trend with only a slight increase (Figure 5A,B). These experiments were carried out under the same experimental conditions but in the presence of CAT, which inhibits protein oxidation. It resulted that, in the presence of CAT, the interaction between liposomes and Syn/DOPAC samples at both protein/catechol ratios and times was partially rescued (Figure 5A,B). Taken together, the results reported above indicate that DOPAC reduces Syn interaction with membranes in a concentration- and time-dependent manner and that oxidation contributes to such reduction. 

Next, we investigated in more detail how oxidation di-per-se and oxidation plus DOPAC affected Syn interaction with negatively charged membranes. The ellipticity at 222 nm of Syn-Ox at time 0 and upon incubation did not change, indicating that oxidized Syn remained unfolded and did not fibrillate [32]. However, the oxidation reduced the ability of Syn to interact with PC:PS membranes. In the presence of the latter, at time 0, the ellipticity at 222 nm of Syn-Ox was slightly higher than that of Syn-Ox alone, and roughly half of that observed for Syn bound to PC:PS. In addition, the signal at 222 nm of Syn-Ox decreased during incubation (168–192 h), indicating that protein oxidation gives rise to species less able to interact with membranes than monomeric Syn-Ox. Thus, DOPAC-induced oxidation can partially explain why the interaction of Syn/D 2x and Syn/D 5x with PC:PS membranes was not as strong as that observed between Syn alone and PC:PS membranes. However, the ability of Syn to interact with PC:PS membranes was recovered upon CAT inhibition of DOPAC-induced oxidation. The recovery was only partial; in fact, the CD signal at 222 nm of Syn-CAT/D 5× recorded in the presence of PC:PS was not strong as that of Syn at T = 0. This is possibly the result of different factors contributing to the decrease of membrane interaction of Syn incubated with DOPAC. In fact, the sample of pre-oxidized Syn incubated with DOPAC (Syn-Ox/D 5x), in particular for 48–72 h, showed a decreased signal at 222 nm, as compared to both Syn-Ox and Syn/D 5x. This finding indicates that, apart from the oxidation mediated by H_2_O_2_ released by DOPAC, Syn-membrane interaction is also modulated directly by the DOPAC effect on Syn. In Figures S7 and S8, the far-UV CD spectra of Syn under the described conditions are reported.

### 2.5. Cellular Uptake and Cytotoxicity of α-Synuclein Aggregates Grown in the Presence of DOPAC

**Figure 6:**
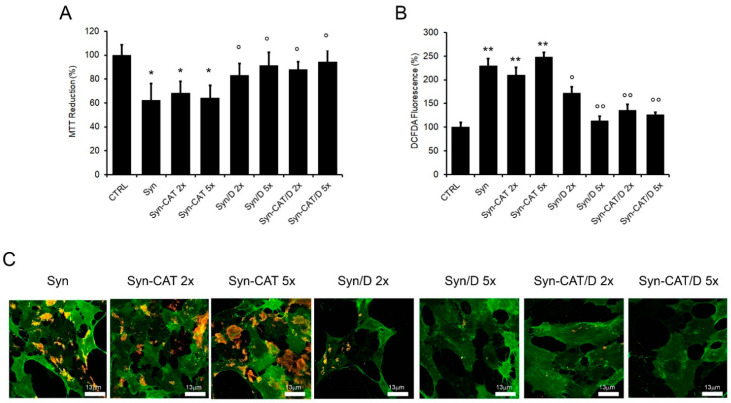
Cytotoxicity of Syn aggregates. Viability of SH-SY5Y cells exposed for 24 h to 5 µM Syn fibrillar aggregates (Syn) grown for 168 h in the absence or in the presence of different protein/CAT, protein/DOPAC or protein/DOPAC + CAT molar ratios (1:2, 1:5). (**A**) MTT assay (**B**) ROS production. Error bars indicate the standard error of three independent experiments carried out in triplicate. * *p* < 0.05, ** *p* < 0.01 vs. control. ° *p* < 0.05; °° *p* < 0.01 vs. Syn aggregates. *p* values are shown in Figure S9. (**C**) Immunolocalization of Syn aggregates on the plasma membrane. The cells were stained with Alexa 488-conjugated CTX-B (green fluorescence); protein aggregates were stained with anti-synuclein antibodies followed by treatment with Alexa 568-conjugated anti-rabbit secondary antibodies (red fluorescence).

Once established the effects of DOPAC on Syn conformational features, aggregation, and aggregate interaction with synthetic membranes, we sought to investigate the biological effects of the Syn-DOPAC complex using the human neuroblastoma cell line SH-SY5Y. The cells were exposed for 48 h to Syn/DOPAC or Syn alone samples in the presence or in the absence of CAT to assay their cytotoxicity in relation to the different protein conformational states. By using the MTT test, we found that Syn samples left in the presence of DOPAC showed scarce cytotoxicity and that cell viability averaged that of control cells (Figure 6A). Accordingly, ROS levels significantly decreased in cells exposed to fibrils grown in the presence of DOPAC (Figure 6B). Eliminating the oxidative events on the protein, caused by DOPAC, by using CAT, we did not observe any differences with respect to the samples treated without CAT. These data suggest that oxidative events are one of the many modifications made by DOPAC but are not responsible for the biological effects of the aggregates (Figure 6A,B).

To further explore the mechanism of DOPAC protection, the interaction of Syn aggregates with the plasma membrane of neuroblastoma cells was monitored by confocal microscopy (Figure 6C). As previously reported [31,37], a large number of Syn aggregates (stained in red) were bound to the cell membrane GM1 (monosialotetrahexosylganglioside 1, stained in green), a widespread cell surface ganglioside, that is a key interaction site for amyloids [41] (Figure 6C). It is important to note that, when the cells were exposed to Syn aggregates grown in the presence of DOPAC or DOPAC and catalase, the presence of aggregates on the cell membranes was drastically reduced in a DOPAC dose-dependent manner (at GM1-enriched sites Figure 6C). However, under the same conditions, Syn aggregates grown in the presence of CAT were found to adhere to the cell membrane. Taken together, these data suggest that the presence of DOPAC during Syn aggregation decreases the presence of the resulting aggregates on the plasma membrane and in turn decreases ROS production and cytotoxicity. 

**Figure 7:**
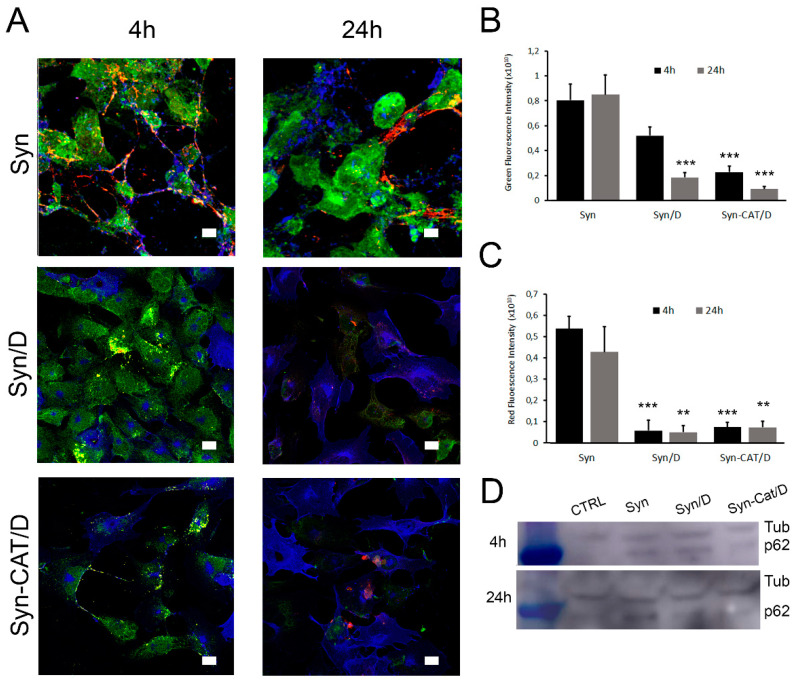
Lysosomal activity in SH-SY5Y cells. SH-SY5Y cells were exposed to 5 µM Syn aggregates grown in the absence (Syn) or in the presence of DOPAC (Syn/D) or DOPAC + CAT (Syn-CAT/D) for 4 h and 24 h. (**A**) Immunofluorescence of cells. Cell membranes were stained with Alexa 488-conjugated CTX-B (blue fluorescence), protein aggregates were stained with anti-synuclein antibodies followed by treatment with Alexa 568-conjugated anti-rabbit secondary antibodies (red fluorescence), and endogenous p62 was stained using the anti-SQSTM1/p62 primary antibodies followed by treatment with Alexa 6333-conjugated anti-mouse secondary antibodies (green fluorescence). Scale bar 13 µm (**B**,**C**) Quantification of green (**B**) and red (**C**) mean fluorescence intensity of almost 10 different acquisitions. (**D**) Western blots of p62 and βTubulin (Tub) used as normalized protein. Two-way ANOVA Test: *** *p* < 0.001 vs. Syn treatment; ** *p* < 0.01 vs. Syn treatment Values are the average ± SE of 10 independent acquisitions.

These data induced us to investigate other cellular mechanisms of aggregates removal. Indeed, the cytotoxicity of Syn aggregates correlates not only with their ability to interact with cell membranes, but also with their uptake and intracellular localization (Figure 7). Previous data have indicated that amyloid fibrils, but not monomers, are taken up by cells in a lysosome-dependent process and that this internalization correlates with fibrils cytotoxicity [42]. Considering this aspect and the reduced presence on the cell membrane of Syn/DOPAC aggregates, we sought to investigate lysosome involvement in our experimental model by assessing the levels of p62, a protein also known as sequestosome 1 (SQSTM1), by confocal microscopy. In lysosomal-dependent processes such as autophagy, p62 usually is used as an indicator of the degradative processes involved in the autophagic flux. Indeed, p62 levels increase during autophagosome formation (the initial step of protein cargo recognition) and decrease during the formation of autophago-lysosomes (degradation step) while an accumulation of this marker occurs if autophagy is deficient [43]. Here, we treated SH-SY5Y cells for 4 h and 24 h with (i) Syn fibrils (aged 168 h), (ii) Syn aggregates grown in the presence of DOPAC 5x (Syn/D) and (iii) Syn species grown for 168 h in the presence of DOPAC 5x and CAT (Syn-CAT/D). We found that cell treatment with Syn samples induced a persistent increase of the p62 levels (green signals) at the two times analyzed (Figure 7A,B). In addition, the noticeable lack of autophagy-mediated clearance of aggregates with a concomitant increase in p62 suggests a deficiency of functional autophagy confirming the cytotoxic effect of this sample previously shown in Figure 6 by MTT assay. The cell treatment with Syn/D induced an initial increase of p62 levels at the shortest time of cells treatment (4 h) and a subsequent decrease at 24 h, as shown by the quantification analysis (Figure 7B) and according also to the Western-blotting images (Figure 7D). This observation and the reduced signals of Syn aggregates (red) suggest the induction of the autophagy process with lysosomal degradation and clearance of Syn aggregates (Figure 7C). These data agree with the recovery of cell viability in the MTT experiments.

## 3. Discussion

Misfolding and subsequent aggregation of Syn following its overexpression, reduced clearance and/or the presence of point mutations are critical hallmarks in PD associated with the loss of nigrostriatal dopaminergic neurons. Any alteration of Syn proteostasis, increased oxidative stress due to mitochondrial dysfunction, altered levels of dopamine metabolites, lipid dysmetabolism increase the likelihood of developing PD [44]. At the present, the administration of the dopamine precursor L-DOPA remains the gold standard for PD and Parkinsonism treatment, even though its continuative use leads to motor complications and drug-induced dyskinesia [45]. New drug targets and new pharmacological molecules are today intensively studied. The knowledge of the structural details of Syn interactions with specific molecules and their relation to altered cellular conditions is of utmost importance to understand their physiological role and design new drugs able to relieve efficiently PD pathogenesis. Here, we studied the in vitro interaction between Syn and DOPAC to understand the correlation between the DOPAC-induced conformational effects on Syn and the new biological properties of the protein. Previously, we studied the molecular interaction of Syn with Oleuropein and DOPET [31,37]. Both molecules exhibited the property to inhibit Syn in the formation of amyloid mature fibrils. The mechanism of this inhibition is not fully understood; therefore, the study of a new molecule that has a part of the chemical structure in common with the previous ones is particularly useful for investigating this question. Furthermore, DOPAC is a harmless metabolite of DOPAL and has previously been shown to be able to interfere with Syn aggregation [30].

**Figure 8:**
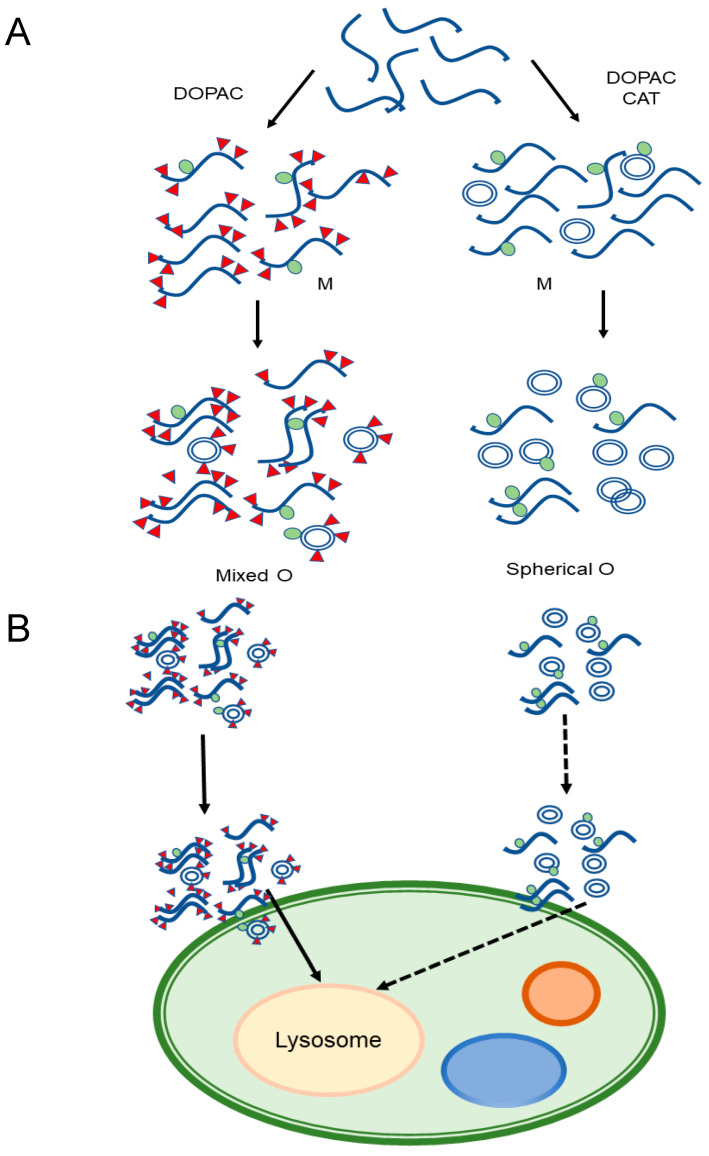
Proposed mechanism of interaction of DOPAC-induced oligomers with cell membranes. In the presence of DOPAC, chemically modified, oxidized aggregated species with different morphologies accumulate. Adding CAT, TEM reveals a population of ring-like oligomers. In both cases, dimers and trimers in equilibrium with monomers are detected by SEC (**A**). Syn/DOPAC aggregates do not undergo conformational changes upon interaction with membrane and could be internalized and degraded by a lysosomal-dependent pathway (**B**).

Our data converge on a description of the DOPAC-induced Syn species as a kind of catechol-protected and shielded oligomer population (Figure 8A), whereby their inherent structure is responsible for their overall biological and biophysical effects. Although Syn does not undergo a change in its secondary structure following the interaction with DOPAC, it experiences substantial physical and chemical changes which converge in inhibition of its fibrillation. The major effect of DOPAC on Syn seems to be a stable oligomerization of the protein. As indicated by the gel filtration and SDS-PAGE data, dimers and trimers begin to form, and their production appears to be linked to protein incubation time and catechol concentration. However, the major component at the used protein/DOPAC ratio is a species eluting as the monomer. At the same time, the protein is chemically modified in terms of oxidation of the methionine residues and for the formation of a covalent adduct on the side chains of the lysine residues. We tried to evaluate these effects to understand which most correlates with the inhibition of Syn aggregation and with the other effects induced by DOPAC, including the reduced propensity to acquire regular secondary structure in the presence of lipid vesicles, and relative stability to proteolysis. Moreover, DOPAC-induced species are not toxic to cells, do not induce ROS formation and exhibit the ability to trigger autophagy. 

The autoxidation product of DOPAC (H_2_O_2_) and the derivative (DOPAC quinone) are responsible for the chemical modifications on Syn, such as extensive oxidation of Met residues and the formation of a covalent adduct likely involving Lys residues [20]. The role of oxidation was considered very important for the Syn aggregation process since it can, by itself, induce Syn oligomerization and inhibit Syn fibrillation [32]. To shed light on the molecular aspects of the interaction between Syn and DOPAC, especially in relation to the protein interaction with the cell membrane and cytotoxicity, Met oxidation to Met-sulfoxide was inhibited by using catalase. Intriguingly, a population of annular oligomers not forming fibrils and not toxic to cells was still detected by hampering oxidation. Therefore, we concluded that oxidation does not inhibit fibril growth by itself but could play additive roles. We also investigated the interaction of oligomeric Syn with lipid membranes and found that, differently from Syn, DOPAC-induced oligomers scarcely underwent conformational transition upon interaction with the latter. The lack of this effect could arise from chemical modifications and/or the oligomerization state. Indeed, in the case of oxidized Syn, two crucial factors for Syn-lipid interaction are no more available: Syn *N*-terminal Met residues (1 and 5) and one of the Lys residues in the *N*-terminal/central region. According to Cholak et al. [46] and others [7,47], the *N*-terminal Met triggers the initial event leading to the cooperative formation of the α-helical structure with lipid membranes. In our scenario, the interaction with membranes for the oxidized and non-oxidized Syn appears to be different. Syn species grown in the presence of DOPAC undergo a scarce conformational modification in the presence of PC:PS membranes, suggesting that the absence of the initial interaction event plays a crucial role in the acquisition of the secondary structure. On the other hand, such interaction is partially recovered in the presence of CAT, although it appears reduced respect to that of the native protein. Therefore, our findings indicate that oxidation is not the only determinant event for the modulation of Syn-membrane interaction. 

The chemical modification of Lys residues was seen also upon Syn reaction with DOPAL and in this case, it involved a large number of Lys residues and was considered a main responsible for the toxicity of the modified protein and a contributing element to PD development [48]. By others, it was also observed that a further reaction of this adduct could lead to the formation of cross-linked dimers and trimers [49]. Here, the number of modified residues did not exceed one per molecule, as shown by MS, and probably not the same modification site is involved, due to the unfolded conformation of the protein, thus it is likely that Syn populations containing different modified Lys residues did coexist. Moreover, in our experiments, we did not detect the presence of covalent dimeric and trimeric forms. Therefore, we think that the modification on the lysine cannot play a decisive role in the reduced Syn folding at the lipid membrane. Depending on Lys position in the sequence, due to different steric hindrance and charge effect, Syn molecules could exhibit different propensity to acquire α-helical structure. 

In conclusion, it is more likely that the formation of DOPAC-induced Syn conformers is the key event responsible for the new properties of the protein. Moreover, we found that the species grown in the presence of DOPAC and CAT were different from the morphological point of view; in fact, ring-shaped aggregates were imaged by TEM in all analyzed samples, while the species containing also oxidations appeared less structured. Therefore, the polymerization products arising in the presence of DOPAC play a stabilizing role and confer to Syn new properties in terms of solubility, reactivity, and stability to proteolysis. It could be that DOPAC acts as a crowding effector, shielding protein monomers and oligomers, conferring protection to proteolysis. Based on this view, it is speculated that DOPAC in addition to induce covalent modifications, can shield the hydrophobic surfaces of monomers and intermediate oligomeric species, preventing the direct interaction between non-polar patches of toxic oligomers, responsible for fibrillation process and cell membrane binding and toxicity. 

Accordingly, Syn aggregates obtained in the presence of DOPAC were also analyzed in terms of cytotoxicity to SH-SY5Y cells. Our data highlight that DOPAC reduced significantly Syn toxicity with respect to Syn fibrils, in terms of MTT reduction and ROS production. These results agree with the immunofluorescence data, where a reduced presence of DOPAC/aggregates on the cell membrane was imaged. Amyloid binding to the membrane results in membrane functional and/or structural perturbation, with derangement of cell signaling [50,51,52] and alterations of free Ca^2+^ and ROS levels [53,54]. Other effects, such as the interaction with membrane receptors [54,55,56,57] and the interference with signaling pathways have also been reported [56,58]. Syn accumulation has been shown to trigger neurotoxicity through aggregation-dependent mechanisms also in Gaucher disease, a severe neurological lysosomal storage disease, a group of metabolic diseases caused by lysosomal dysfunction [59,60,61,62]. Under this aspect, Gaucher disease recalls the widely reported decline of cellular degradative functions, specifically the autophagy-lysosomal pathway, in neurodegenerative disorders, including PD [63]. In this context, we analyzed the lysosomal activity by monitoring the p62 levels in cells exposed to Syn aggregates grown in the absence or in the presence of DOPAC. We found involvement of lysosomal accumulation after cell treatment with Syn fibrils, in agreement with previous data [42], whereas Syn/DOPAC small aggregates were internalized and degraded through a lysosomal-dependent pathway. This observation was confirmed by the reduction of p62 levels and by the presence of the aggregates on the cell membrane. Overall, these findings and other recent data suggest that the major mechanisms for the degradation of exogenous and endogenous Syn fibrils are mediated by lysosomal activity and provide crucial support for better understanding cell-to-cell spread of Syn inclusions as a function of the normal cellular proteostasis mechanism designed to remove protein aggregates from the cell.

Summing up, DOPAC-induced Syn aggregates stay only temporarily on the surface of the cell, successively are internalized quite fast into the cell, maybe through receptors present on the membrane (Figure 8B). Once inside the cell, they do not alter cellular viability, induce lysosomal process and subsequent autophagy, in analogy to Syn fibrils [64], with the ensuing delay of the progression of PD and similar diseases. The proteolysis hypothesis of PD considers that the loss of nigrostriatal neurons is primarily associated with the strong tendency to aggregate and to form toxic oligomers by Syn. During neurodegeneration, both DOPAL and the DOPAL/DOPAC ratio increase [65] and Syn abnormally accumulates [66], thus increasing the chance of oligomer formation. In turn, a raised concentration of oligomers increases the fraction of oligomers undergoing internalization, eventually triggering lysosome activity and autophagy. In the oligomeric form, Syn crosses the cell membrane, possibly by a receptor-mediated mechanism. The outcome of the event depends on whether the internalized species are toxic, as in the case of DOPAL-induced oligomers, or not [67]. Otherwise, the toxicity could be due to the aldehyde moiety that being highly reactive could still react with amine-containing molecules forming Schiff base and not for the DOPAL-induced Syn intrinsic structural properties. Through an analysis of molecular interaction between Syn and DOPAC, our study converges towards a unifying view of DOPAC (catechol)-induced Syn species and hopefully provides useful information for the future design of drugs for PD-targeted therapies.

## 4. Materials and Methods

### 4.1. Materials

3,4-Dihydroxyphenylacetic acid (DOPAC) and lyophilized bovine catalase (CAT) were purchased from Merck (Darmstadt, Germany). Phosphatidylserine (PS) and Phosphatidylcholine (PC) were purchased from Avanti Polar Lipids (Alabaster, AL, USA). All reagents and chemicals used for cell culture, obtained from Sigma or Fluka (St. Louis, MO, USA) were of analytical reagent grade.

### 4.2. Expression and Purification of Recombinant Human α-Synuclein

Human Syn was expressed in E. coli BL21 (DE3) cell line transfected with the pT7–7/α-syn plasmid. Overexpression of the protein was achieved by growing cells in LB medium at 37 °C to an A600 nm of 0.6, followed by induction with 0.5 mM isopropyl β-thiogalactopyranoside. The purification of the recombinant protein was conducted following a procedure previously described [68], and further purified by RP-HPLC. Protein identity and integrity were assessed by mass spectrometry (MS).

### 4.3. α-Synuclein Aggregation In Vitro

Recombinant lyophilized Syn was dissolved in 20 mM sodium phosphate buffer, pH 7.4, and then filtered through a 0.22 μm PVDF membrane (Millipore, Bedford, MA, USA). Protein concentration was estimated by UV spectroscopy and then adjusted to a final concentration of 70 μM (1.0 mg/mL) in all samples. An Eppendorf thermomixer Compact (Eppendorf, Hamburg, Germany) was employed to carry out in vitro aggregation. Centrifuge tubes containing the protein solution were incubated for up to 7 days (168 h) at 37 °C under shaking at 750 rpm. Two sets of experiments were performed: the first one involved Syn and DOPAC at different molar ratios (1:2, 1:5); the second one included the use of bovine catalase (CAT). A fresh 1.0 mg/mL catalase solution prepared in 50 mM potassium phosphate buffer, pH 7.0, was diluted in order to obtain the amount of catalase needed for each sample in a volume of 1.0 μL. ThT fluorescence assay [69] was used to monitor fibril formation during the aggregation experiments. A Cary Eclipse fluorescence spectrophotometer (Agilent Technologies, Santa Clara, CA, USA) was used to measure dye emission collected in the 455–600 nm range, after excitation at 440 nm, of a freshly prepared 25 μM ThT solution in 25 mM sodium phosphate buffer, pH 6.0, filtered with a 0.22 μm PES membrane, at a final protein concentration of 60 μg/mL.

### 4.4. Chromatographic and Mass Spectrometry Analysis

Size exclusion chromatography was carried out in an AKTA FPLC System (Amersham Biosciences, Uppsala, Sweden) with a Superdex™ 200 Increase 10/300 GL column (GE Healthcare Bio-Sciences AB, Uppsala, Sweden). Sample aliquots (100 μL) were withdrawn from the aggregation mixture, centrifuged, loaded onto the column, and eluted at 0.75 mL/min in 20 mM Tris-HCl buffer, pH 7.4, containing 0.15 M NaCl. The detector was set at 214 nm. Column calibration was obtained loading a mixture of proteins with known molecular weight (Thyroglobulin, Apoferritin, Bovine Serum Albumin, Ovalbumin, Carbonic Anhydrase, Ribonuclease A, Aprotinin). Reverse Phase (RP)-HPLC analyses were carried out on a 1200 series Agilent Technologies (Santa Clara, CA, USA) chromatographer, using a Jupiter C18 column (4.6 mm × 250 mm, 5.0 μm; Phenomenex, Torrence, CA, USA). The column was run with a water/acetonitrile gradient containing 0.1% trifluoroacetic acid, (5% to 38% in 5 min, 38% to 43% in 15 min) at a wavelength of 226 nm. For fingerprinting analysis, a different gradient (5% to 25% in 5 min, 25% to 28% in 13 min, 28% to 39% in 3 min, 39% to 45% in 21 min) was used. In this analysis, the effluent was monitored by recording the absorbance at 226 nm and 340 nm. All chromatographic analyses were performed in triplicate. Sample identity was assessed by MS using a Xevo^®^ G2-XS ESI-Q-TOF mass spectrometer (Waters Corporation, Milford, MA, USA). Measurements were conducted in positive ion mode, using a capillary potential of 1.5 kV. For native MS, samples were dialyzed in 200 mM ammonium acetate, pH 7.0. The analyses were performed in nanoflow mode by using quartz emitters produced in-house by using a Sutter Instruments Co. (Novato, CA, USA) P2000 laser pipette puller. Up to 5.0 µL samples were typically loaded onto each emitter by using a gel-loader pipette tip. A stainless-steel wire was inserted in the back-end of the emitter to supply an ionizing voltage in the range of 1–1.6 kV. Source temperature was set at 30 °C, desolvation voltage was 40 V. 

### 4.5. Electrophoresis

SDS-PAGE was performed on a 13% T, 2.6% C, pH 8.8, acrylamide separating gel according to [70]. The electrophoretic run was performed at room temperature with a starting current intensity of 10 mA/slab, and then 12 mA/slab. The gels were stained with a Coomassie Blue 250 solution and destained with an ethanol:acetic acid:water solution.

### 4.6. Proteolysis of α-Synuclein Species

Limited proteolysis of Syn/DOPAC samples was carried out in 20 mM sodium phosphate buffer, pH 7.4, at room temperature using proteinase K (PK) at an enzyme to substrate (E/S) ratio of 1:1000 (by weight) and 70 μM Syn concentration. The reactions were quenched after 5–15 min by adding TFA in water (0.2%, *v/v*) and analyzed by RP-HPLC. 

### 4.7. Transmission Electron Microscopy (TEM) Analysis

All analyses were performed by negative staining method, in which 5.0 μL of sample was diluted to 0.25 mg/mL of protein; the sample was dried on the plastic grid and subsequently stained with 10 μL of 1.0% (*w/v*) uranyl acetate solution. A Tecnai G2 12 Twin TEM microscope (FEI Company, Hillsboro, OR, USA) for sample imaging. Size distribution of protein samples was calculated on 200 particles manually extracted from the micrographs using ImageJ software. Only clearly defined spherical and isolated particles were selected. The species with diameters larger than 100 nm were excluded. Measurements were obtained with a standard deviation of ±8.

### 4.8. Structural Characterization

The conformational changes of Syn were monitored in the far-UV in the 250–196 nm range by a Jasco J-710 spectropolarimeter (Tokyo, Japan). All analyses were carried out in a 1.0 mm path length quartz cuvette, in which the samples were loaded at a concentration of 0.1 μg/μL in 25 mM sodium phosphate buffer, pH 7.4. Spectra acquisition was performed using the following parameters: data pitch of 0.2 nm; continuous scanning mode at 20 nm/min; response of 8 s; band-width of 2.0 nm; 3 accumulations. The mean residue ellipticity [θ] (degree cm^2^ dmol^−1^) was calculated from the formula [θ] = (θ_obs_/10) × (MRW/lc), where θ_obs_ is the observed ellipticity in degrees, MRW is the mean residue molecular weight of the protein, l the optical path length in cm, and c is protein concentration in g/mL.

### 4.9. Interaction with Lipid Membranes

Small unilamellar vesicles (SUV) made with phosphatidylcholine (PC) and phosphatidylserine (PS) were obtained using a 1:1 lipid ratio. The mixture was dried in N_2_ atmosphere and, then, put under a high vacuum for 90 min to remove residual chloroform. Sodium phosphate buffer was added to a final phospholipid concentration of 6.0 mM, and five cycles of freeze and thaw under mixing were performed after 1 h to allow bilayer formation. The extrusion of lipid suspension by Liposofast device, using a 0.1 μm membrane was performed for an odd number of steps (11) to ensure removal of the starting multilamellar vesicles. The product was stored at 4 °C before use. The mean diameter of 1:1 PC:PS vesicles was ~120 nm, with a good PDI index monitored by dynamic light scattering.

### 4.10. Cell Culture Conditions and Treatments

SH-SY5Y cells were cultured at 37 °C in a humidified incubator under a 5.0% CO_2_ atmosphere in 50% HAM/50% DMEM culture medium supplemented with 10% fetal bovine serum, 3.0 mM glutamine, 100 units/mL penicillin and 100 μg/mL streptomycin. The cells were exposed for 48 h to vehicle or to 5.0 μM Syn samples. 

### 4.11. Cell Viability

The MTT reagent (3-(4,5-dimethylthiazol-2-yl)- 2,5-diphenyltetrazolium bromide (Sigma-Aldrich, St. Louis, MO, USA) was added to SH-SY5Y cells seeded in 96-well plates (1 × 104 cell/well) at 0.5 mg/mL final concentration. After incubation for 2 h at 37 °C, formazan crystals formed from MTT by the reducing activity of mitochondrial dehydrogenases were dissolved with 100 μL of solubilization solution and the absorbance was measured at 570 nm in a microplate reader (Bio-Rad, Hercules, CA, USA). Non-viable cells were unable to reduce MTT. Final absorption was calculated by averaging three independent measurements of each sample after subtraction of the average of the blank solution (100 μL of MTT solution and 100 μL of lysis buffer: 20% SDS, 50% *N*,*N*-dimethylformamide).

### 4.12. Reactive Oxygen Species Measurements

The probe 2′,7′–dichlorofluorescein diacetate, acetyl ester (CM-H2 DCFDA; Sigma-Aldrich) was added at a 10 mM final concentration to SH-SY5Y cells plated in 96-well plates (1 × 104 cell/well). After 30 min, fluorescence values were read at 538 nm by Fluoroscan Ascent FL (Thermo-Fisher, Illkinch, France).

### 4.13. Confocal Immunofluorescence

Subconfluent SH-SY5Y cells grown on glass coverslips were treated for 48 h with Syn aggregates at a 5.0 μM final concentration (monomeric protein concentration) and then washed with PBS Cell membranes were stained with 10 ng/mL CTX-B Alexa488 (GM1 membrane staining, green channel). Syn and p62 proteins were stained with rabbit polyclonal anti-Syn antibodies (1:500) (Abcam, Cambridge, UK) and with mouse polyclonal anti SQSTM1/p62 (1:500) followed by the Alexa Fluor 568-conjugated secondary antibodies (red channel) or by Alexa Fluo 633-conjugated secondary antibodies (far-red channel), respectively. Cell fluorescence was imaged using a confocal Leica TCS SP8 scanning microscope (Leica, Mannheim, DE, USA). The observations were done using a Leica HC PL Apo CS2 X63-oil immersion objective. Images and signal fluorescence were composed and analyzed by Image J Fiji software. 

### 4.14. Western Blotting

SH-SY5Y cells (105 cells/well) were plated in a 6-well plate for 24 h. Following the different treatments, the cells were washed with PBS and then lysed in 100 μL of 1× Laemmli buffer (62.5 mM Tris-HCl buffer, pH 6.8, 10% (*w/v*) SDS, 25% (*w/v*) glycerol) without bromophenol blue. Whole-cell lysates were collected and boiled at 95 °C for 5 min and then centrifuged at 12,000× *g* for 5 min at 4 °C. Total protein concentration in lysates was measured by the BCA protein assay kit. β-mercaptoethanol and bromophenol blue were added to an equal amount of protein (20 μg) from each sample, whose components were separated in precast SDS-PAGE gels (Biorad #456-8096) and then transferred onto nitrocellulose membrane by Trans-Blot Turbo Transfer Pack (Biorad #1704157). The immunoblots were incubated at room temperature in PBS containing 5.0% (*w/v*) bovine serum albumin, 0.1% (*v/v*) Tween 20 and probed with primary and appropriate secondary antibodies. The antibody used in immunoblotting was specific for p62 (Abcam) and for β-Tubulin (Santa-Cruz). Finally, the membranes were repeatedly washed in 0.5% (*v/v*) PBS-Tween^®^-20 solution and protein bands were detected using the Clarity Western ECL solution. Chemiluminescent signals were acquired by using AmershamTM 600 Imager imaging system (GE Healthcare Life Science, Pittsburgh, PA, USA).

### 4.15. Statistics

Data were expressed as mean ± standard error and the statistical significance was evaluated by two-way analysis of variance (ANOVA). 

## Figures and Tables

**Table 1:** Molecular masses of the main protein species purified by RP-HPLC from Syn and Syn/DOPAC samples, after 48 and 168 h of incubation. DOPAC reaction during the ionization in ESI source (below). The carboxyl moiety (blue) is lost under the MS conditions used. Structure of the proposed adduct according to the found molecular weight by MS analysis (below, right). In notes, 48 h and 168 h indicate the time of incubation when the species were present in the mixture. The star on the structure indicates the site of binding with Syn.

Sample	RP-HPLC RT (min)	Molecular Mass (Da)		Protein Species	Notes
		Found	Calculated		
Syn	25.1	14,460.21 ± 0.01	14,460.19	Syn	48 h–168 h
Syn/DOPAC	16.0	168.19 ± 0.09	168.15	DOPAC	48 h–168 h
	16.5	123.00 ± 0.01	123.0	DOPAC derivative	Generated in MS source [36]
	22.5	14,524.71 ± 0.11	14,524.19	Syn + 4 ox	168 h
	23.2	14,507.68 ± 0.03	14,508.19	Syn + 3 ox	48 h–168 h
	24.1	14,492.51 ± 0.03	14,492.19	Syn + 2 ox	48 h–168 h
	25.1	14,460.57 ± 0.05	14,460.19	Syn	48 h
	33.2	14,581.75 ± 0.55	14,581.41	Syn + 121	Oxidized and modified Syn was also found in this fraction
Syn/DOPAC + CAT	25.1	14,460.27 ± 0.02	14,460.19	Syn	48 h
	33.2	14,581.41 ± 0.32	14,581.19	Syn + 121	48 h–168 h
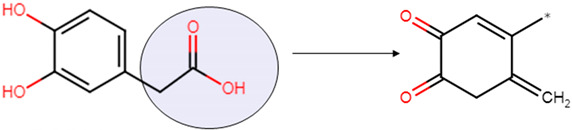					

**Table 2:** Molecular masses of fragments produced by limited proteolysis with PK of Syn species obtained by SEC (Figure 4, inset). Proteolysis was performed for 5- and 10-min of incubation of Syn species with the protease. Peptides were purified by RP-HPLC (Figure 4). Oligomer (T + D) + PK, Figure 4A; Monomer, M + PK, Figure 4B; Syn + PK, Figure 4C. The sign + or − indicates if the fragment absorbs or not at 340 nm.

Sample	RP-HPLC RT (min)	Molecular Mass (Da)		Protein Species	Absorbance at 340 nm
		Found	Calculated		
Oligomer (T + D)	19	5185.09 ± 0.37	5185.51	95–140	-
	19.6	538.41 ± 0.30	5389.74	93–140	-
		5632.71 ± 0.42	5633.00	90–140	-
	27.6	7173.53 ± 0.21	7173.74	73–140	-
	29.5	8815.42 ± 0.16	8815.58	57–140	+
		9086.56 ± 0.91	9086.90	54–140	
	31.1	11,413.18 ± 0.41	11,412.59	31–140	+
		12,597.78 ± 0.09	12,596.91	19–140	
Monomer (M)	11–15	Polymeric and aggregated DOPAC derived species			
	19	5185.02 ± 0.30	5185.51	95–140	-
	19.6	5389.31 ± 0.20	5389.74	93–140	-
		5632.78 ± 0.49	5633.0	90–140	-
	27.6	7173.58 ± 0.50	7173.74	73–140	-
	28.5	5662.26 ± 0.02	5662.62	1–56	+
		5783.93 ± 1.02	5783.62	1–56 + 121	+
	28.9	7305.50 ± 0.99	7304.46	1–72	+
	29.1	7930.98 ± 0.32	7931.17	1–79	+
	29.5	8815.52 ± 0.53	8815.58	57–140	+
		9087.50 ± 1.25	9086.90	54–140	
	30.5	8845.17 ± 1.85	8845.19	1–89	+
	31.4	9086.52 ± 0.50	9086.90	54–140	+
	31.2	11,413.38 ± 0.11	11,412.59	31–140	-
		12,597.32 ± 0.19	12,596.91	19–140	-
	34.5	14,460.32 ± 0.51	14,460.19	1–140	-
Syn	13.8	3148.59 ± 0.21	3148.19	114–140	-
	19.0	5185.02 ± 0.30	5185.51	95–140	-
	19.6	538.31 ± 0.20	5389.74	93–140	-
		5632.78 ± 0.49	5633.0	90–140	-
	27.6	7173.92 ± 0.29	7173.74	73–140	-
	28.4	6810.75 ± 0.02	6811.86	5–72	-
	28.6	5662.08 ± 0.60	5662.62	1–56	-
	28.9	7304.07 ± 0.09	7304.46	1–72	-
	29.1	7930.33 ± 0.75	7931.17	1–79	-
	29.5	7923.04 ± 0.81	7923.02	9–89	-
	30.4	8845.11 ± 0.49	8845.19	1–89	-
	31.1	9088.53 ± 1.27	9086.90	54–140	-
	34.5	14,460.32 ± 0.51	14,460.19	1–140	-

## Data Availability

Not applicable.

## Supplementary Materials

The following are available online at https://www.mdpi.com/article/10.3390/ijms22116008/s1.
