# Supplementary material for: Structural Features and Toxicity of α-Synuclein Oligomers Grown in the Presence of DOPAC
Source: Int J Mol Sci. 2021 Jun 2;22(11):6008. doi: 10.3390/ijms22116008 (PMC8199589; doi:10.3390/ijms22116008)
Supplement: Supplementary file 1 [file ijms-22-06008-s001.zip › ijms-1203291-supplementary.pdf]

International Journal of
Molecular Sciences

MDPI

Supplementary figures

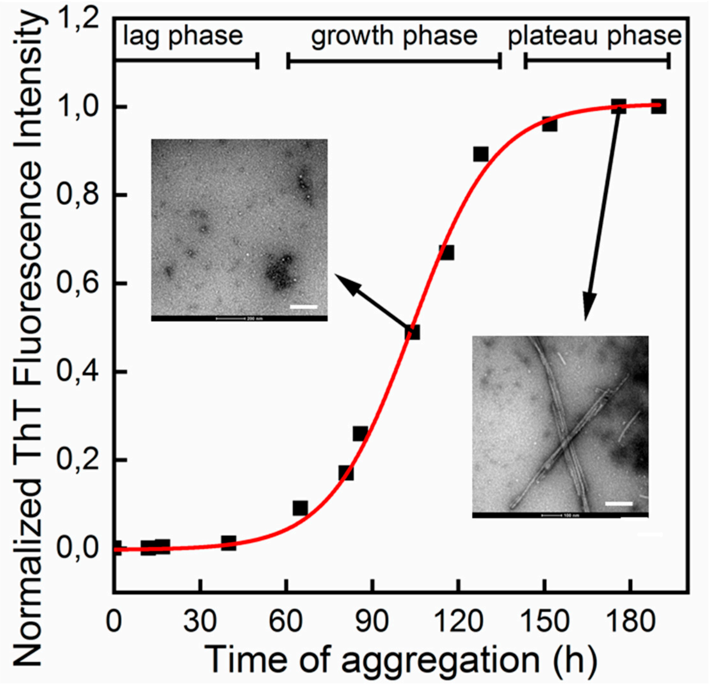

Figure S1. Aggregation process of Syn monitored by ThT assay. The intensity of ThT fluorescence at 485 nm was recorded after excitation at 440 nm for Syn samples collected by the aggregation mixture at the indicated time points. Inset: TEM pictures (500 nm of magnification) of Syn samples corresponding to 95 (left) and 168 (right) hours of incubation

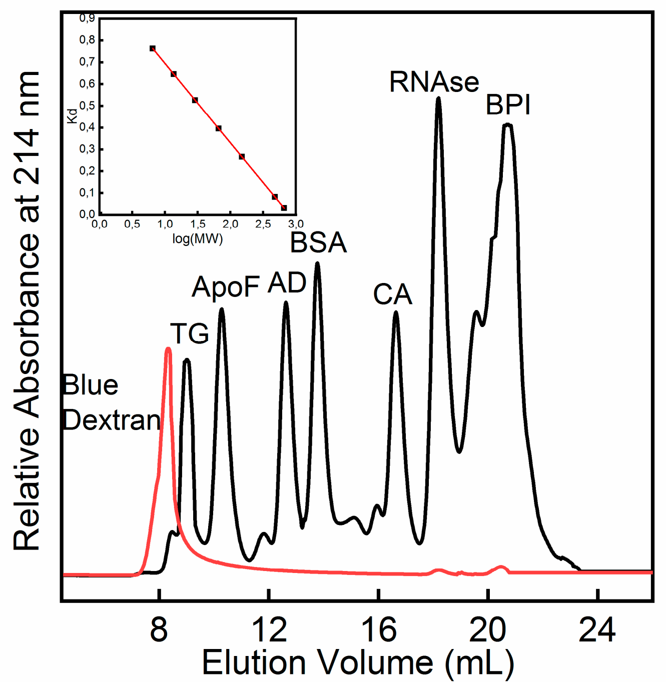

Figure S2. SEC calibration. SEC analysis of proteins of known molecular weight for the calibration of the Superdex™ 200 Increase 10/300 GL column (GE Healthcare Bio-Sciences AB, Uppsala, Sweden). The column was eluted with 20 mM Tris-HCl buffer, pH 7.4, containing 0.15 M NaCl at 0.75 mL/min. The detector was set at 214 nm. The following species were used: Thyroglobulin (TG), Apoferritin (ApoF), Alcohol Dehydrogenase (AD), Bovine Serum Albumin (BSA), Carbonic Anhydrases (CA), Ribonuclease (RNAase), Aprotinin (BPI). The red line represents the analysis of Blue Dextran, used to calculate the dead volume of the column. Inset: the calibration line (red) expressing the correlation between Log (MW) of the species and their Kd by the equation y = -0.365x + 1.0597.

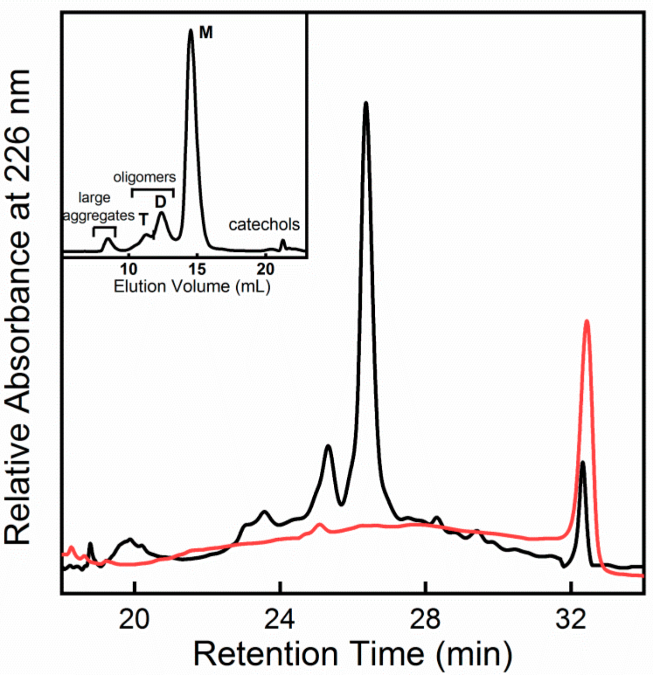

Figure S3. RP-HPLC chromatograms of the fractions from SEC (inset) corresponding to dimer and trimer (D+T, red line) and monomer (M, black line). The analyses were carried out on a 1200 series Agilent Technologies (Santa Clara, California, USA), using a Jupiter C18 column (4.6 mm x 250 mm, 5 µm; Phenomenex, CA, USA). The runs were done with the gradient of water and acetonitrile, containing 0.1% of TFA, from 5 to 38% in 5 min and from 38 to 43% in 15 min at a wavelength of 226 nm. The SEC profile refers to the analysis s of Syn/DOPAC (1:5) samples after 48 h of incubation in the presence of CAT.

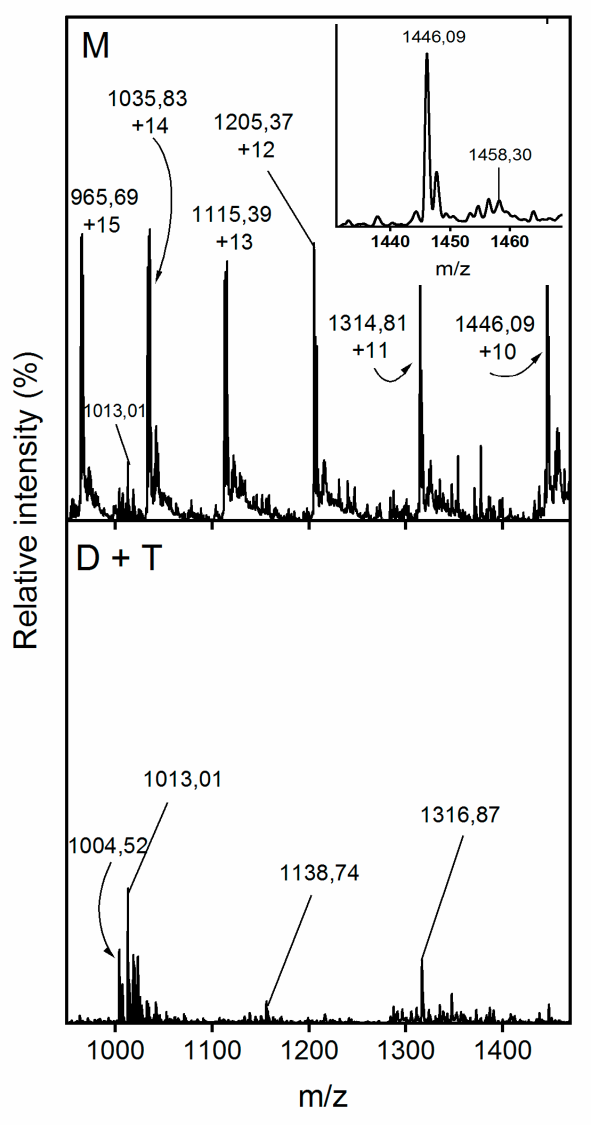

Figure S4. Native mass spectrometry. Mass/charge spectra of monomeric (M) and oligomeric (D + T) Syn fractions from SEC. Inset: zoom of m/z spectrum from 1420 to 1470.

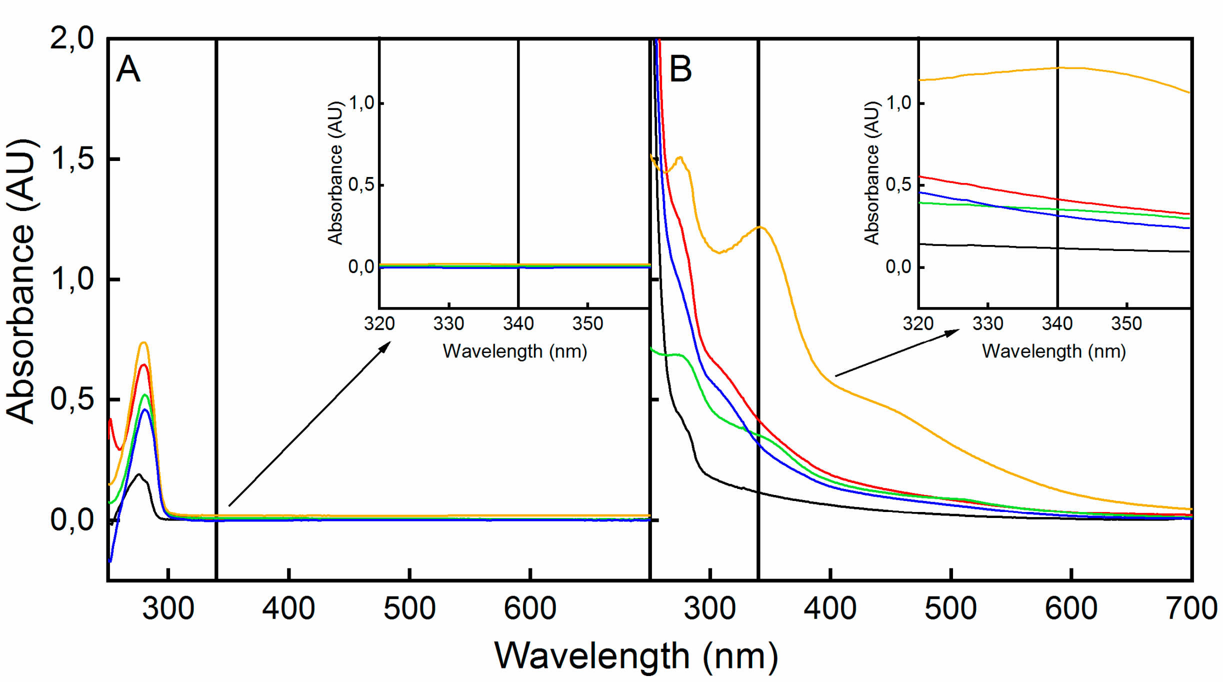

Figure S5. Spectroscopic analysis. UV-visible spectra of DOPAC and Syn just solubilized (A) and after 168 h of incubation (B). The curves refer to Syn (black line), Syn/DOPAC (1:5, red line), Syn/DOPAC (1:5) in the presence of CAT (brown line), DOPAC (blue line) and DOPAC with CAT (green line). Inset: zoom of the UV-vis spectra in the range 320-360 nm.

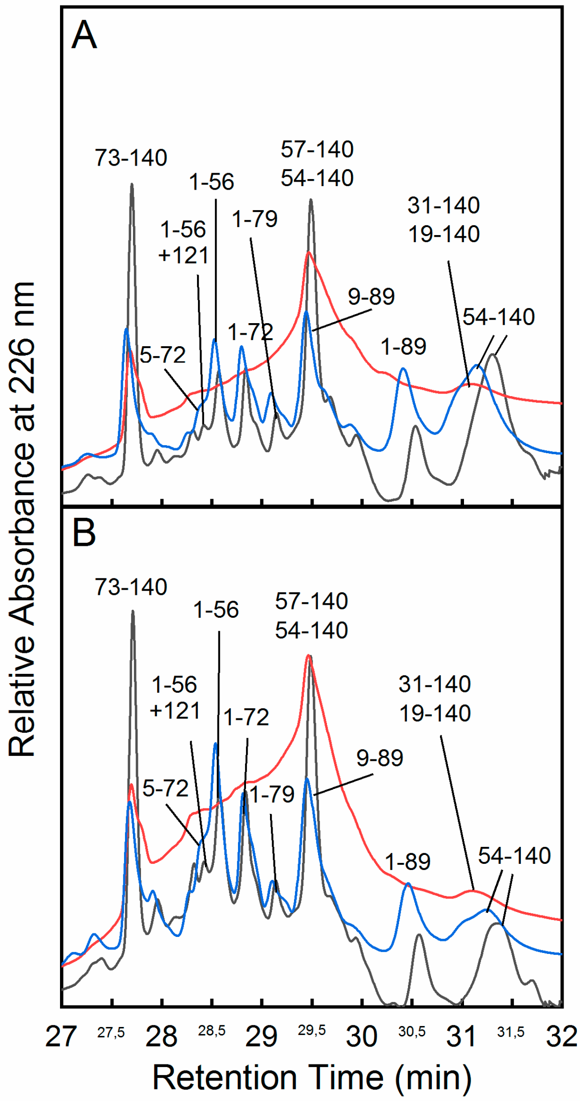

Figure S6. RP-HPLC analysis. Zoom from 27 to 32 minutes of the RP-HPLC chromatograms of the proteolytic mixtures of the SEC fractions corresponding to dimer and trimer (oligomer, red line), monomer (black line) and Syn (blue line) with PK (Fig. 4). Aliquots were collected from the reaction mixtures after 5 (A) and 10 (B) minutes of incubation with the protease and analyzed monitoring the signals at 226 nm. The proteolysis reactions were conducted by using an enzyme to protein ratio of 1:1000. RP-HPLC conditions were reported in the experimental section. The numbers close to the peaks correspond to the fragments identified by MS analysis (Table 2).

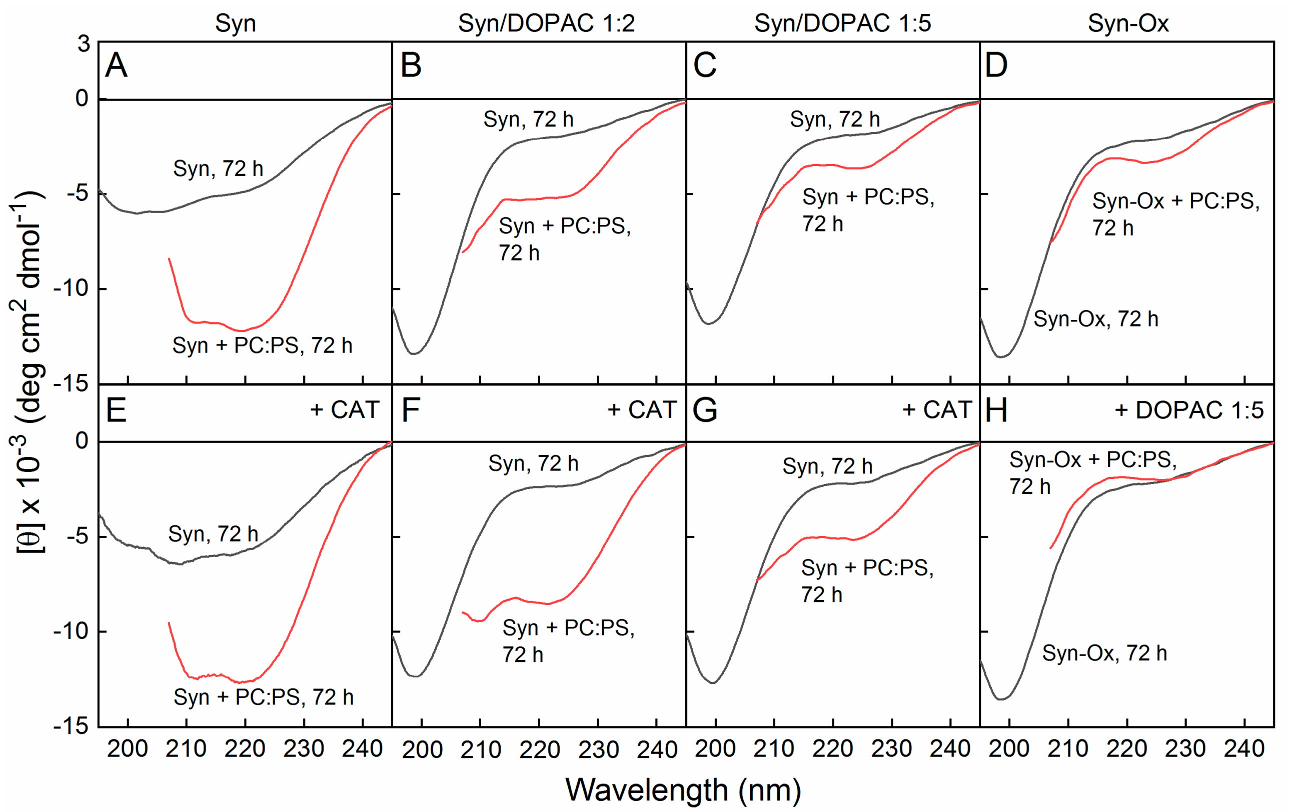

Figure S7. Far-UV CD spectra of Syn in the presence of membrane after 72 h of incubation. Spectra in black are obtained in the absence of PC:PS membranes, those in red in their presence. The spectra in B, C, D, E are relative to Syn samples containing DOPAC (1:2, B,F; 1:5, C,G). The spectra in E, F, G are recorded in the presence of CAT. In D and F the spectra of oxidized Syn are reported in the absence (D) and in the presence (H) of DOPAC. The experimental details are described in Methods.

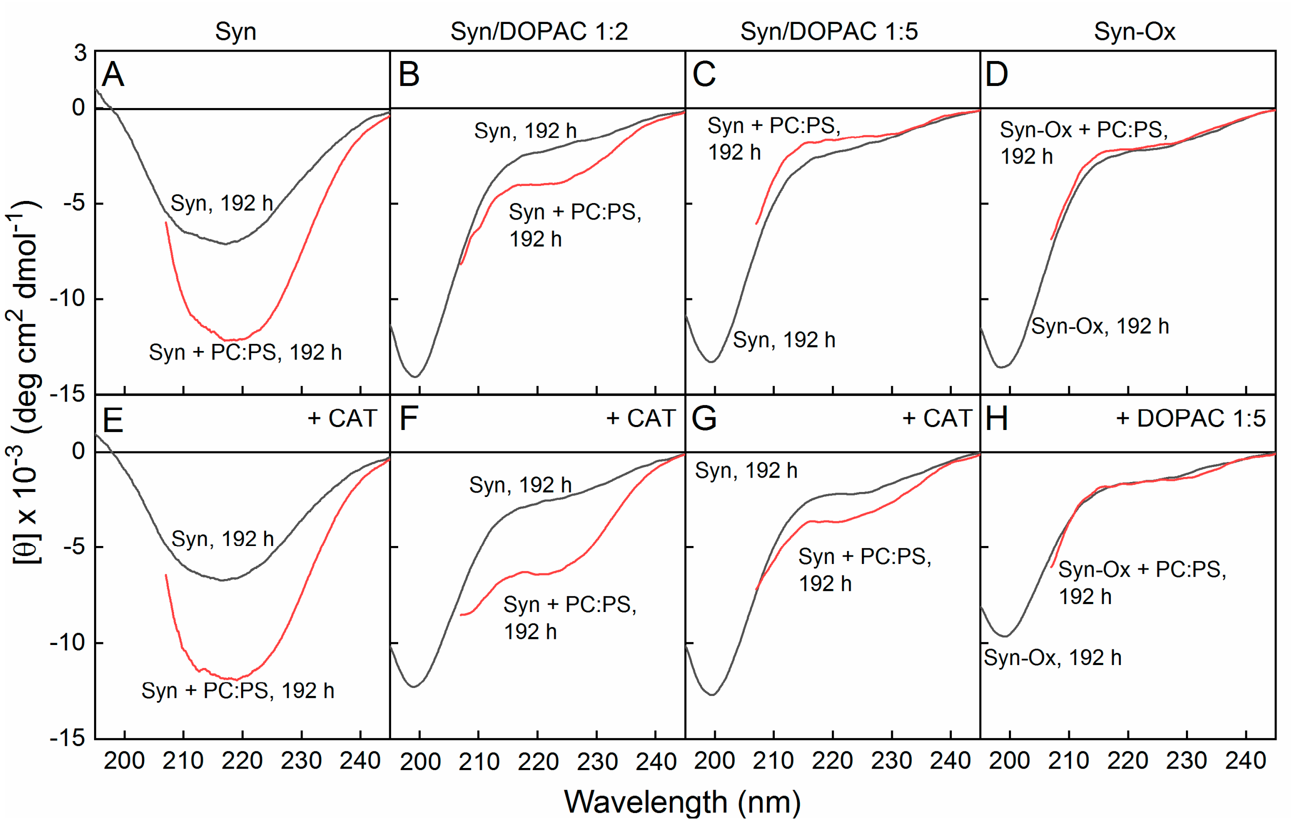

Figure S8. Far-UV CD spectra of Syn in the presence of membrane after 192 h of incubation. Spectra in black are obtained in the absence of PC:PS membranes, those in red in their presence. The spectra in B, C, D, E are relative to Syn samples containing DOPAC (1:2, B,F; 1:5, C,G). The spectra in E, F, G are recorded in the presence of CAT. In D and F the spectra of oxidized Syn are reported in the absence (D) and in the presence (H) of DOPAC. The experimental details are described in Methods.

Comparison	p Value two-way ANOVA	
	MTT	ROS
CTRL vs Syn	0.048	0.0087
CTRLvs Syn-Cat2x	0.037	0.0062
CTRL vs Syn-Cat5x	0.024	0.0082
CTRL vs Syn/D2x	0.996	0.278
CTRL vs Syn/D5x	0.954	0.231
CTRL vs Syn-Cat/D2x	0.0972	0.624
CTRL vs Syn-Cat/D5x	0.999	0.495
Syn vs Syn-Cat2x	1	0.796
Syn vs Syn-Cat5x	0.9793	0.8194
Syn vs Syn/D2x	0.037	0.0453
Syn vs Syn/D5x	0.028	0.0065
Syn vs Syn-Cat/D2x	0.0183	0.0037
Syn vs Syn-Cat/D5x	0.037	0.029

Comparisons	p Value two-way Anova	
**Green**	**4h**	**24h**
Syn vs Syn/D	0.07	<0.0001
Syn vs Syn-Cat/D	<0.0001	<0.0001
**Red**		
Syn vs Syn/D	<0.0001	0.001
Syn vs Syn-Cat/D	<0.0001	0.002

Figure S9. Statistical analysis for MTT assay and ROS assay (Fig. 6) and for quantification analysis of fluorescence (Fig. 7). Up. Estimated p values of the two-way analysis of variance (ANOVA) for experimental results of MTT and ROS assays reported in Figure 6. Down. Estimated p values of the two-way analysis of variance (ANOVA) for experimental results of quantified signals obtained by immunofluorescence images reported in Figure 7.
